# Inhibin B in healthy and cryptorchid boys

**DOI:** 10.1186/s13052-018-0523-8

**Published:** 2018-07-16

**Authors:** Susanna Esposito, Marta Cofini, Donato Rigante, Alberto Leonardi, Laura Lucchetti, Clelia Cipolla, Lucia Lanciotti, Laura Penta

**Affiliations:** 1 Pediatric Clinic, Department of Surgical and Biomedical Sciences, Università degli Studi di Perugia, Piazza Menghini 1, 06129 Perugia, Italy; 2 Institute of Pediatrics, Università Cattolica Sacro Cuore, Fondazione Policlinico Universitario A. Gemelli, Rome, Italy

**Keywords:** Inhibin B, Cryptorchidism, Puberty, Child

## Abstract

**Background:**

Cryptorchidism, the most common male genital abnormality observed in paediatrics, might often be associated with long-term functional consequences and can even reoccur after a successful orchidopexy. Serum markers that identify cryptorchid boys with gonadal dysfunction early should be useful in a decision-making process. Inhibin B, produced during all of childhood but altered in cryptorchid subjects, appears strictly related to Sertoli cells, and its levels directly reflect the status of the testis germinative epithelium. Unfortunately, its precise roles in bilateral and unilateral cryptorchidism are still debated and being unravelled. Herein, we report the most current knowledge about inhibin B in both healthy boys and those with cryptorchidism to discuss and clarify its potential clinical applications.

**Discussion:**

Inhibin B represents a simple and repeatable serum marker and it seems to well asses the presence and function of the testicular tissue. Testicular tissue in prepubertal age is largely made up of Sertoli cells; inhibin B, coming from working Sertoli cells, allows to indirectly evaluate their function. Besides, inhibin B is produced throughout childhood, even before puberty, in contrast with central hormones, and it is not influenced by androgens during puberty, in contrast with other testicular hormones. Although further studies are needed, low levels of inhibin B have been related with low testicular score and/or with consistent alterations of testicular parameters at histological examination. This means that inhibin B could be an indirect marker of testicular functions that could even replace testicular biopsies, but current data are inconsistent to confirm this potential role of inhibin B in cryptorchidism.

**Conclusion:**

Inhibin B represents an effective candidate for early identification of testicular dysfunction after orchidopexy for cryptorchidism. Unfortunately, current data cannot exactly clarify the real role of inhibin B as a predictor of future testicular function in cryptorchidism and future long-term follow-up studies, with repeated inhibin B checks both in cryptorchid and in formerly cryptorchid children and adolescents, will permit to assess if previous normal levels of inhibin B would match with future normal pubertal development and fertility potential.

## Background

Cryptorchidism is the most common abnormality of male sexual development, characterized by impaired descent of one or both testes into the scrotum [1]; it occurs in 2–4% of full-term boys [2]. This incidence increases in preterm neonates, especially if they also have low birth weight [3]. The exact mechanisms behind cryptorchidism are still not completely understood and different causes lead to different levels of impaired testicular functions and fertility potential. Descent of testes into the scrotum results from a multifactorial process, involving anatomic structure and hormonal functions. In the intraabdominal phase insulin-like hormone 3 seems to play a prior role [1]. This hormone is directly produced by Leydig cells; therefore every alteration in their function, occurring during the foetal stage, might lead to development of cryptorchidism [4]. Instead inguino-scrotal phase is completely under androgen control, so bilateral cryptorchidism could be an expression of hypogonadotropic hypogonadism [5, 6], or of an insufficient androgen production or of an androgen insensitivity [7].

In general terms, cryptorchidism may occur as an isolated abnormality or be associated with other anatomic alterations of male genitalia, such as hypospadias and micropenis [1] or as an expression of testicular dysgenesis syndrome [4]. Recent evidence suggests that exposure to smoking, organochlorines, or phthalate could negatively affect gonadal development, as could maternal diabetes, including pregestational and gestational forms [6, 8]. Bilateral cryptorchidism represents a frequent feature of children with atypical genitalia [9]. It also could be a part of systemic syndromes involving multiple organs, such as Noonan [10], Prader-Willi [1], VATER/VACTERL [11], Prune-Belly [12], and CHARGE syndromes [13] and RASopathies [14].

Human and animal models have shown that histological and cytological alterations found in undescended testes could be largely attributed to high temperature exposure, due to anatomic malposition, or to environmental or maternal factors occurring during pregnancy, rather than to primitive testicular dysfunction [15–19].

The majority of children with cryptorchidism could experience spontaneous testis descent within the first 6 months of life, but in the remaining cases, orchidopexy is recommended in the first 18 months to preserve normal testicular germ cell maturation [20]. Notably, there is a proportional decrease in sperm count as the age at which orchidopexy is performed increases: 76% of children who undergo surgery between 10 months and 4 years have a normal sperm count, while only 26% do if surgery is postponed between 4 and 14 years [21]. Furthermore, the number of germ cells is widely decreased in cryptorchid testes operated on at 3 years relative to those operated on at 9 months [22].

The fertility failure occurs in about 38% of subjects with bilateral cryptorchidism [23], approximately 10% of those with unilateral cryptorchidism [24] and only 5% of non-cryptorchid males [23]. Bilateral cryptorchid testes seem to have a lower number of germ cells, and bilateral cryptorchidism is often associated with degenerative changes in the testes involving both germ and Sertoli cells [25, 26].

Children with cryptorchidism seem to have a different hormonal pattern than the general healthy population, so a reliable serum marker that identifies pre-pubertal boys with abnormal gonadal function early could be useful because its precocious assessment would allow to select patients with high risk to future impaired testicular functions and to establish a correct follow-up program [27].

Neither follicle stimulating hormone (FSH) nor luteinizing hormone (LH) seems to be a reliable marker of testicular function in cryptorchid boys. Indeed both FSH and LH may be increased in cryptorchid children, due to loss of physiologic feed-back, or to primitive hypergonadotropic hypogonadism [28, 29], rarely that conditions cannot be diagnosed in prepubertal age for the transient inactivation of the hypothalamus hypothalamus gonads axis [30]. Interestingly in boys with nonmosaic Klinefelter syndrome there was a higher peak of FSH during post-natal activation of hypothalamus hypothalamus gonads axis than in normal children, associated with normal levels of inhibin B [31]. These data might suggest a precocious altered sensitivity of Sertoli cells to FSH stimulation.

Furthermore, as previously assessed, cryptorchidism may represent the expression of hypogonadotropic hypogonadism [5, 6].

Inhibin B is not influenced by androgen secretion during puberty and it is so related to Sertoli cells, independently of their maturation status. Consequently, it seems to be more useful than for example anti-müllerian hormone (AMH) for long-term follow-up of cryptorchid subjects trough infancy to adolescents. AMH is associated particularly with the immature Sertoli cells and is affected by the inhibitory action of androgens in puberty because only mature cells have androgen receptors [32]. Furthermore, inhibin B could be useful also for any pubertal cryptorchidism.

Many studies have stressed a possible role of inhibin B as a marker of testicular function. Frequently inhibin B serum levels are decreased in cryptorchid children and in formerly cryptorchid men, in both bilateral and unilateral forms [28, 29, 33, 34]. Unfortunately, little is known about the exact role of inhibin B in childhood, and their practical use is still debated [35–38].

In this review, we have discussed the most recent knowledge about inhibin B in childhood, their alterations in cryptorchid children and adolescents, and their correlations with testicular parameters to clarify a possible application in the management of cryptorchidism. A MedLine research via PubMed was undertaken to identify studies using the following terms as keywords: “inhibin B and cryptorchidism”, “inhibin B and puberty”, “cryptorchidism and puberty”, “inhibin B and testicular parameters”, “inhibin B and childhood”. We have reviewed all papers published in the last 10 years, and the date of our last search was September 1st 2017. Only English language articles were analysed, all references were consulted, and relevant articles were included.

## Discussion

### Inhibin B in boys during childhood and puberty

Inhibin B is a heterodimeric glycoprotein hormone belonging to the superfamily of growth β factors and consists of one α and one β subunits [36]. The production of inhibin B seems different before and after puberty, in both animal and human models: in prepubertal testes, it is related only to Sertoli cells, which are able to produce both α and β units [4], while after puberty it can be produced also by germ cells [38]. In puberal boys inhibin B increases immediately after the increase of serum FSH, suggesting that FSH could stimulates Sertoli cells [39]. Prepubertal Sertoli cells are able to produce inhibin B also following stimulation with human chorionic gonadotropin (HCG), while this cannot happen in more mature Sertoli cells [40].

### Inhibin B in cryptorchid children

The serum levels of inhibin B are strictly related to the germinative epithelium status. They seem to directly reflect Sertoli cells functions [41] and to be directly related to the number of spermatogonia even in cryptorchid subjects [42]. Indeed inhibin B serum levels result very low in cryptorchid children and young boys [17, 41, 42]. In children and young boys with cryptorchid, a low inhibin B/FSH ratio was also detected [17, 42], often associated with increased levels of FSH in children with spontaneous descent of both testes and in those affected by “mild” forms of cryptorchidism. These data suggest that even spontaneously descended testes and “mild” undescended testes might show some testicular dysfunction [17].

After bilateral orchidopexy, low levels of inhibin B could be suspicious for hypogonadotropic hypogonadism, particularly if associated with decreased levels of FSH [41].

In children with cryptorchidism, the inhibin B response after a short course of HCG changed at different ages: it increased only in prepubertal boys, while no response or even a decrease was found in older ones. Therefore, hormonal response patterns seem different depending on the maturation level of the testicular tissue [40].

As previously stated, inhibin B is produced under FSH stimulation. However, the real role of this relationship in the development of a normal testicular function is still unknown and its possible role in childhood remains controversial. For example, Raivio et al. demonstrated that this negative relation may be already present in prepubertal boys with cryptorchidism underwent to HCG therapy, even under the age of 2 years. Hormonal sample in these subjects demonstrated suppression of FSH associated with increase of inhibin B; this could be due to a not yet well understood interaction between Sertoli cells, Leydig cells and germ cells [43]. This negative relationship was confirmed in a more recent study [41] and seems to start after the first 6 months of life in children bilateral cryptorchidism [44].

In contrast Chada et colleagues have found a positive relation between FSH and inhibin B during male minipuberty, confirming the importance of increase the knowledge about this crucial stage of gonadal development [45].

However animal model (adult male monkeys) have demonstrated that the feed-back of inhibin B on FSH results stronger than the feed-forward of FSH on inhibin B [46].

### Inhibin B values: relationship between unilateral and bilateral cryptorchidism

In a study on 62 cryptorchid prepubertal boys (17 had a bilateral form and 45 unilateral), no difference was found in serum concentrations of FSH, LH, inhibin B, testosterone or sex hormone-binding globulin between cryptorchid subjects and a control group. No differences were found also between unilateral and bilateral cryptorchidism. The only difference was a lower level of inhibin B in bilateral cryptorchidism with one or both testes non-palpable when compared with bilateral cryptorchidism with both testes palpable at clinical examination. After adjustment for age, only in severe forms of bilateral cryptorchidism low levels inhibin B serum levels were found [40].

Thorup et al. have also evaluated inhibin B as a marker of the presence and function of testicular tissue. Indeed, boys with bilateral cryptorchidism showed higher levels of inhibin B, associated with lower FSH and LH levels, than boys with bilateral vanished testes. However, even in this paper, all hormonal serum levels tested outside the normal range in cryptorchid subjects [47]. In contrast, the same work group did not find any difference between unilateral cryptorchidism and unilateral vanished testis in terms of inhibin B levels: this may be due to some degree of contralateral testicular compensation [48]. The possible importance of testicular position was stressed also by comparing children with both testes palpable at the superficial inguinal ring with unilateral cryptorchidism: inhibin B was decrease in the first group, even after adjustment for age [49].

In a very recent published study, serum levels of inhibin B of 27 boys with bilateral and unilateral cryptorchidism were evaluated and compared with control groups of the same age range (mean age 26.6 and 24.2 months respectively). After adjustment for age, inhibin B resulted lower in cryptorchid boys than in control group, especially for subjects with bilateral cryptorchidism [50].

In contrast, previous data reported no relation between testis position and hormonal patterns. Moreover, no differences were found between unilateral and bilateral cases and there was a positive relationship between inhibin B and the number of A-dark spermatogonia. The number of A-dark spermatogonia seemed to be related to normal number of tubular germ cells, normal FSH and LH serum levels and normal inhibin B levels [50]. Longui et al. had first demonstrated a relationship between inhibin B and the number of spermatogonia, assessing inhibin B serum levels and testicular biopsy after HCG stimulation in cryptorchid boys less than 4 years of age: inhibin B values were significantly related to the mean number of spermatogonia [42].

Cortes et al. found positive link between LH and inhibin B, assessing that it is mandatory for the correct development of A-dark spermatogonia from gonocytes even in prepubertal cryptorchid children [51]. Indeed, previous data had reported the importance of this positive association only in the pubertal age [35]. The existence of this association and the true role in spermatogenesis must be confirmed by further studies.

A more recent study has investigated histological and endocrine hormonal status of 71 boys (age range from 7 months to 5.4 years) underwent to orchidopexy for cryptorchidism (24% with bilateral and 76% with unilateral forms). Histological parameters involved the number of tubules with spermatogonia compared with the total number counted tubules, the number of A-dark spermatogonia and the total number of A-dark spermatogonia compared to the total number of counted tubules (minimal number considered was 100 tubules); while hormonal sample included serum levels of FSH, LH and inhibin B. No significant association was found between inhibin B levels and histological findings at the testicular biopsy [52, 53].

Studies that have related inhibin B to bilateral versus unilateral cryptorchidism are shown in Table 1.

**Table 1:** Inhibin B values in cryptorchid subjects in relationship between unilateral and bilateral cryptorchidism

Study group	Subjects investigated	Results
*Zivkovic* et al.	Unilateral vs bilateral with testes at the superficial inguinal ring	Inhibin B was higher in unilateral cryptorchidism
*Thorup* et al.	Bilateral cryptorchidism vs bilateral vanished testes	Inhibin B was higher in cryptorchid subjects in combination with lower values of FSH and LH
*Thorup* et al.	Unilateral cryptorchidism vs unilateral vanished testes	No difference between the two groups in terms of inhibin B, FSH or LH
*Cortes* et al.	Unilateral and bilateral cryptorchid subjects	Low inhibin B in bilateral cryptorchid boys with testes at superficial inguinal ring than unilateral forms
*Hamdi* et al	Unilateral and bilateral cryptorchid subjects compared with control group	Low inhibin B in cryptorchid boys, especially in bilateral forms

### Inhibin B values: relationship with testicular parameters

Cortes et al. compared inhibin B and FSH serum levels with testicular parameters obtained through testicular biopsy in children with bilateral cryptorchidism: all subjects showed low concentrations of spermatogonia and gonocytes at biopsy, nearly 24% also had low levels of inhibin B and 9% of boys with both these parameters also had increased FSH, while a decreased level of FSH was found in about 5%, compared to normal ranges for age. The authors concluded that low inhibin B levels could be directly related to impaired testicular function [41].

Thorup et al. have investigated gonadotropin and inhibin B levels and number of germ cells at biopsy in cryptorchid children before and after surgery to better define the future fertility outcome: good fertility after orchidopexy was associated with normal levels of inhibin B, normal numbers of germ cells and normal for age levels FSH and LH, which were high before surgery. Children with lack of normalization of FSH and LH and with no increase of inhibin B after surgery had some degree of testicular dysfunction, while children with normal levels of FSH and LH associated with decreased germ cell number showed transient hypothalamus-pituitary-gonadal dysfunction and consequently a decrease in fertility potential [47].

### Inhibin B values: relationship with orchidopexy

Irkilata et al. analysed children with bilateral cryptorchidism before orchidopexy and at 6 months after surgery, comparing hormone levels with data from testicular biopsies. Twenty-seven boys were included in the study, all of them were undergone to inguinal orchidopexy and in 15 also testicular biopsy was performed. Boys with successful orchidopexy had a statistically significant increase of inhibin B serum levels at 6 months evaluation, suggesting a positive effect of surgery on Sertoli cell development [54]. Testicular tissue in prepubertal age is largerly made up of Sertioli cells [55], confirming the importance of this cellular type for physiological future functions of testes.In this study, the lack of increase of inhibin B serum levels after surgery was related with low testicular score at testicular biopsy [56]. Testicular score was defined as the number of spermatogonia per tubular transverse section and the percentage of tubular transverse section containing spermatogonia, calculating by spermatogonia count in 100 seminiferous tubules in transverse section. These results could confirm the direct association between inhibin B and testicular functions. Unfortunately, testicular biopsies were performed only in few patients [54] and this is not enough to firmly assess that low levels inhibin B is a sure and reliable serum marker of low testicular functions.

In a study a significantly increased level of inhibin B was observed after classic inguinal orchidopexy and after scrotal incision orchidopexy only in those boys 2–9 years old with a unilateral undescended testes [57]. In boys whose surgery was performed at age 0–18 months or 10–12 years, the increase was not reported to be statistically significant. Moreover, in the 18 boys with bilateral undescended testes, inhibin-B levels were unchanged 6 months after surgery [56].

A large randomized controlled study has analysed testicular development and gonadal hormones in boys with bilateral or unilateral cryptorchidism before and after orchidopexy performed at 9 months or at 3 years of age. In this study, interestingly, inhibin B showed a bimodal trend: at 2 months of age bilateral cryptorchid boys had higher serum levels if compared with unilateral cryptorchid boys or boys with spontaneously descended testes, while at 4 years inhibin B was lower in boys with persistent unilateral or bilateral cryptorchidism undergoing orchidopexy than in those with previous spontaneous descent of testes. At the 2-month control an increased number of Sertoli cells was found in the bilateral cryptorchid group compared with the unilateral group. Furthermore, in unilateral undescended testes inhibin B was positively related to the number of Sertoli cells in patients underwent to surgery at 9 months, but this relationship was not present in children underwent to surgery at 3 years of age [22].

## Conclusion

Inhibin B represents a simple and repeatable serum marker that seems to well asses the presence and function of the testicular tissue. Consequently, in our opinion it might represent an effective candidate for early identification of testicular dysfunction after orchidopexy for cryptorchidism.

Testicular tissue in prepubertal age is largely made up of Sertoli cells; inhibin B, coming from working Sertoli cells, allows to indirectly evaluate their function. Besides, inhibin B is produced throughout childhood, even before puberty, in contrast with central hormones, such as LH and FSH. Inhibin B is not influenced by androgens during puberty, in contrast with other testicular hormones, such as AMH. Unfortunately, current data cannot exactly clarify the real role of inhibin B as a predictor of future testicular function in cryptorchidism. Low levels of inhibin B have been related with low testicular score and/or with consistent alterations of testicular parameters at histological examination. This means that it could be an indirect marker of testicular functions that could even replace testicular biopsies, but current data do not permit to confirm this potential role of inhibin B in cryptorchidism. However, long-term follow-up studies, with repeated inhibin B checks both in cryptorchid and in formerly cryptorchid children and adolescents, will permit to assess if previous normal levels of inhibin B would match with future normal pubertal development and fertility potential.
